# Architecture and connectivity of the human angular gyrus and of its homolog region in the macaque brain

**DOI:** 10.1007/s00429-022-02509-7

**Published:** 2022-06-13

**Authors:** Meiqi Niu, Nicola Palomero-Gallagher

**Affiliations:** 1 Institute of Neuroscience and Medicine (INM-1), Research Centre Jülich, Jülich, Germany; 2 C. & O. Vogt Institute for Brain Research, Medical Faculty, University Hospital Düsseldorf, Heinrich-Heine-University Düsseldorf, Düsseldorf, Germany; 3 Department of Psychiatry, Psychotherapy, and Psychosomatics, Medical Faculty, RWTH Aachen University, Aachen, Germany

**Keywords:** Homologies, Cytoarchitecture, Myeloarchitecture, Receptorarchitecture, Parcellation, Angular gyrus, Posterior inferior parietal lobe, Connectivity

## Abstract

The angular gyrus roughly corresponds to Brodmann’s area 39, which is a multimodal association brain region located in the posterior apex of the human inferior parietal lobe, at its interface with the temporal and occipital lobes. It encompasses two cyto- and receptor architectonically distinct areas: caudal PGp and rostral PGa. The macaque brain does not present an angular gyrus in the strict sense, and the establishment of homologies was further hindered by the fact that Brodmann defined a single cytoarchitectonic area covering the entire guenon inferior parietal lobule in the monkey brain, i.e. area 7. Latter architectonic studies revealed the existence of 6 architectonically distinct areas within macaque area 7, further connectivity and functional imaging studies supported the hypothesis that the most posterior of these macaque areas, namely Opt and PG, may constitute the homologs of human areas PGp and PGa, respectively. The present review provides an overview of the cyto-, myelo and receptor architecture of human areas PGp and PGa, as well as of their counterparts in the macaque brain, and summarizes current knowledge on the connectivity of these brain areas. Finally, the present study elaborates on the rationale behind the definition of these homologies and their importance in translational studies.

The angular gyrus (AG) is a horseshoe-shaped region of the posterior part of the human inferior parietal lobule (IPL), where it can be seen as a continuation of the upswing of the superior temporal gyrus. Thus, the AG lies between the parietal, occipital, and temporal lobes, and has been functionally characterized as a higher-order associative cortical region which plays a prominent role in the integration of multiple sensory systems, as well as language comprehension, number processing, spatial attention and memory retrieval. (Binkofski et al. 2016; Seghier 2013; also see articles by Bellana et al., Bush and Bonnici, Graves et al., Sokolowski et al., Kuhnke et al., Pinheiro-Chagas and Parvizi, Rusconi, Desai et al., and Zhang et al. in the present Special Issue).

## Gross anatomy of the angular gyrus

**Fig. 1:**
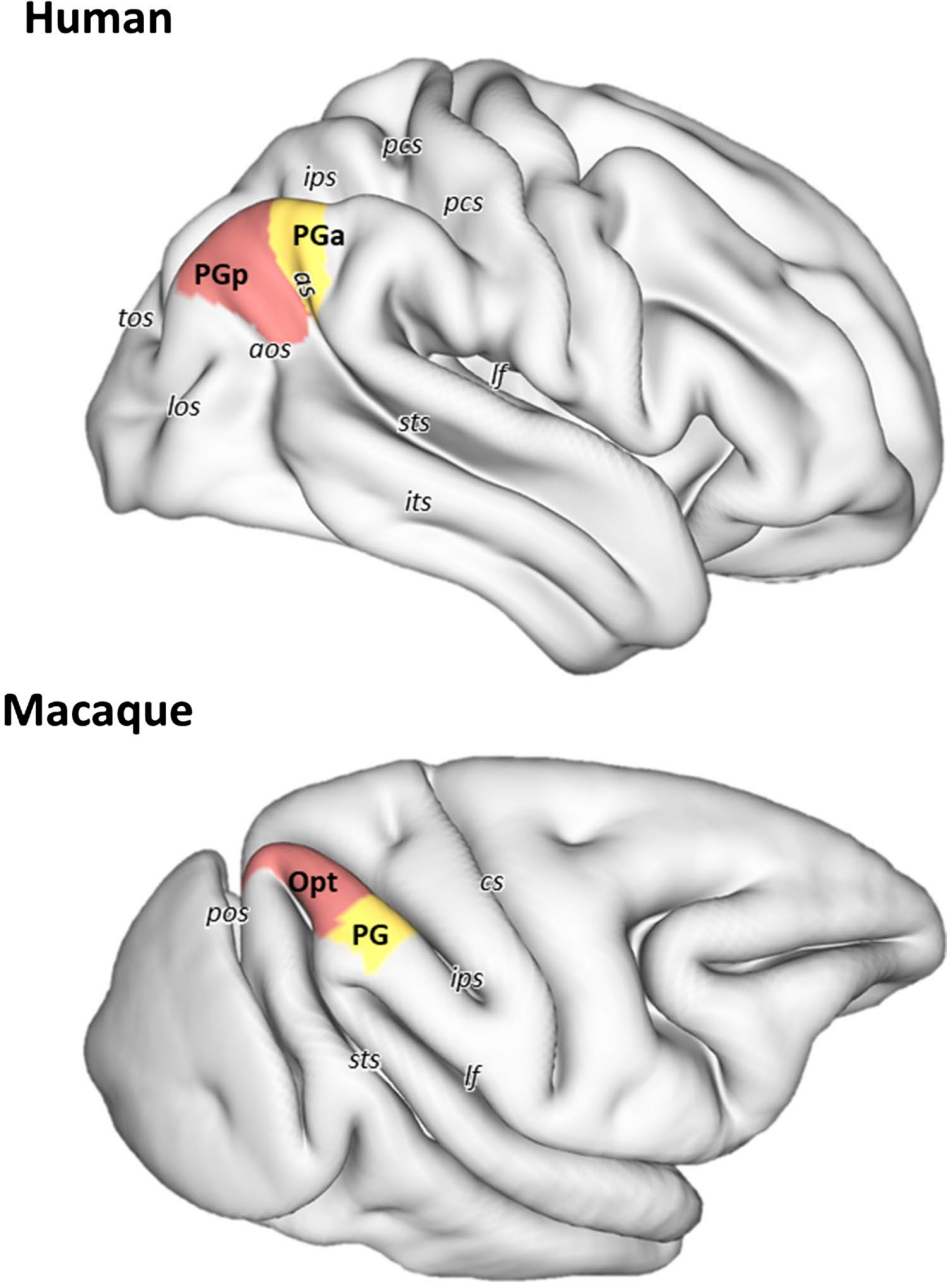
3D reconstruction of the right hemisphere (in lateral view) of human and macaque brains showing the location and extent of the areas that occupy the human angular gyrus and their macaque counterparts. Abbreviations: *pcs* post-central sulcus, *as* angular sulcus, *ips* intraparietal sulcus, *lf* lateral fissure, *sts* superior temporal sulcus, *its* inferior temporal sulcus, *aos* anterior occipital sulcus, *los* lateral occipital sulcus, *tos* transverse occipital sulcus, *cs* central sulcus, *pos* parietal-occipital sulcus

The AG is often described as the gyrus surrounding the angular sulcus (*as*). However, this sulcus is not only highly variable across brains, but also hemispheres (Eidelberg and Galaburda 1984). It arises as the posterior termination of the superior temporal sulcus (*sts*) in 84% of right hemispheres and in 92% of left hemispheres (Ono et al. 1990). In the case of a double parallel *sts*, the superior segment is designated as the *as*. On rare occasions (only in 4% of left hemispheres) the *as* has even been described as a free sulcus (Ono et al. 1990). The dorsal boundary of the AG is roughly located at, or close to, the lateral shoulder of the intraparietal sulcus (*ips*). The AG is abutted rostrally by the supramarginal gyrus (SMG), from which it is separated by the primary intermediate sulcus or sulcus of Jensen. However, this Jensen sulcus is highly variable and only present in 24% of the right hemispheres and 80% of the right hemispheres. Indeed, due to this variability, the sulcus of Jensen is not visible in the HCP S900 surface shown in Fig. 1, since it is a population-based surface and represents the average of 19 brains (Van Essen et al. 2017). The convergence of the posterior end of the *sts* with the anterior occipital sulcus (*aos*) has been described as macroanatomical landmark for the ventral and caudal boundaries, respectively, of the AG (Caspers et al. 2008). However, due to the considerable variability of AG topography, these two sulci are only connected with each other in 52% of the left and 40% of the right hemispheres (Ono et al. 1990). Therefore, there is seldom a consistent topographical landmark which reliably marks the caudal or ventral boundaries with the occipital and temporal lobes, respectively. Indeed, the AG has been described as one of the brain regions with the largest inter-individual variability in morphology (Croxson et al. 2018; Mueller et al. 2013). Given this lack of reliable macroanatomical landmarks, it is not surprising that studies reporting volumetric measurements of the AG present highly differing values (Rademacher et al. 1992).

As a representative brain region involved in high-level integrative cognitive operations (Buckner and Krienen 2013; also see articles by Bellana et al., Bush and Bonnici, Graves et al., Sokolowski h et al., Kuhnke et al., Pinheiro-Chagas and Parvizi, Rusconi, Desai et al., and Zhang et al. in the present Special Issue), the AG is one of the last brain regions to develop in humans (Mars et al. 2011), since it continues maturing into young adulthood (Sotiras et al. 2017). Furthermore, this region is one of the most expanded cortical regions in humans versus non-human primates (Hill et al. 2010; Mars et al. 2011), and previous reports have suggested that striking differences exist in the location and size of this region between humans and non-human primates (Hyvärinen 1982; Seghier 2013). Indeed, in macaque monkeys it is not possible to identify an AG in the strictest sense, not only because macaques present an overall lower degree of gyrification than humans (Zilles et al. 2013), but also because the IPL is one of the region’s brain presenting highest local gyrification levels in the human brain (Jockwitz et al. 2017).

Although it is not easy to define a macroanatomical homolog of AG in the macaque monkey brain, connectivity and functional studies have demonstrated comparable functional domains for the AG in humans and the posterior IPL in macaques (Andersen and Buneo 2002; Caspers et al. 2011; Mountcastle et al. 1975; Orban 2016; also see Vijayakumar et al. in the present Special Issue and further chapters in this article).

## Architectonic parcellations

Microstructural parcellations of the AG can be found in most classical brain maps of the early twentieth century. These studies were based on cyto- or myeloarchitectonic criteria and, with the exception of Vogt and Vogt (Brodmann 1909; Campbell 1905; Smith 1907; Vogt and Vogt 1919), considered the AG to be a homogeneous brain region (Brodmann 1909; Campbell 1905; Flechsig 1920; Smith 1907; von Economo and Koskinas 1925; see Table 1). Latter studies adopted the parcellation schemes of Brodmann (1909) and refined them by defining subdivisions within the described areas (Batsch 1956; Gerhardt 1940; Hopf 1969; Hopf and Vitzthum 1957; Nieuwenhuys et al. 2015; Sarkisov et al. 1949; Schulze et al. 1960). Recently, a quantitative multimodal analysis confirmed that the AG is occupied mainly by two cyto- and receptor architectonically distinct areas, i.e., PGp and PGa (Caspers et al. 2006, 2012), which are now widely used in recent connectivity and functional region-based studies. However, it must be noted that, in some cases, the most anterior portion of the AG is occupied by area PFm, which abuts PGa rostrally (Caspers et al. 2008).

**Table 1:** Comparison of different architectonical parcellation schemes of the human AG and its macaque counterpart

Species	Author	Year	Method	Subdivisions
Human	Campbell	1905	Cyto, myelo	Part of magnopyramidal cortex
	Smith	1907	Cyto	Pari A
	Brodmann	1909	Cyto	BA39
	Vogt	1911	Myelo	90 (on the rostral portion of AG)
	Flechsig	1920	Myelo	42
	Von Economo & Koskinas	1925	Cyto	PG
	Gerhardt	1940	Cyto	89 (posterior part), 90 (anterior part)
	Sarkisov et al.	1955	Cyto	39, 39s
	Batsch	1956	Myelo	90
	Hopf & Vitzthum	1957	Myelo	90
	Schulze et al.	1960	Cyto	90
	Eidelberg & Galaburda	1984	Cyto	PG, PEG, OPG
	Caspers et al.	2006/2012	Cyto, receptor	PGp, PGa
	Nieuwenhuys et al.	2015	Myelo	89 (posterior part), 90
Macaque	Brodmann	1905	Cyto	7 (posterior part)
	Vogt & Vogt	1919	Cyto, myelo	7a
	Von Bonin & Bailey	1947	Cyto	PG
	Pandya & Seltzer	1982	Cyto	Opt, PG
	Andersen et al.	1990	Cyto, myelo	7a
	Preuss & Goldman-Rakic	1991	Cyto, myelo	7a-m, 7a-l
	Lewis & Van Essen	2000	Cyto, myelo, SMI-32	7a
	Geyer et al.	2005	Cyto, 5-HT_1A_	Opt, PG
	Gregorious et al.	2006	Cyto, myelo, SMI-32	Opt, PG
	Niu et al.	2021	Cyto, receptor	Opt, PG

*Cyto* cytoarchitectonic analysis; *myelo* myeloarchitectonic analysis; *receptor* receptor architectonic analysis

In macaque monkeys, the posterior IPL contains the caudal half of Brodmann’s area (BA) 7 (Brodmann 1905), area 7a of Vogt and Vogt (1919), or area PG of Von Bonin and Bailey (1947). Later on, Pandya and Seltzer (1982) and Preuss and Goldman-Rakic (1991) identified cytoarchitectonic differences between the parietal convexity and the parietal operculum. The former discerned two cytoarchitectonic divisions along the posterior inferior parietal convexity (caudal area Opt and rostral area PG). The latter separated this region into a dorsal area 7a-m (on the rim of the intraparietal sulcus) and a ventral area 7a-l (on the free surface). The map of Pandya and Seltzer (1982) was later confirmed by a combined cyto- and myeloarchitectonic analysis (Gregoriou et al. 2006), as well as by differences in the distribution patterns of multiple receptors (Niu et al. 2021), and dominates the current view on the structural organization of the macaque caudal IPL.

Specifically, a homology between human areas PGp and PGa with macaque areas Opt and PG, respectively, is now widely accepted (Caspers et al. 2011; Gregoriou et al. 2006; Mars et al. 2011; Niu et al. 2021; Patel et al. 2019; Petrides and Pandya 2009). Thus, in the following chapters we will describe the cyto-, myelo- and receptor architecture, as well as the connectivity patterns of these areas. For each modality, we will emphasize the characteristics which support the hypothesis of these homologies between humans and macaque monkeys.

## Cyto-, myelo- and receptor architectonic features

### Cytoarchitecture

**Fig. 2:**
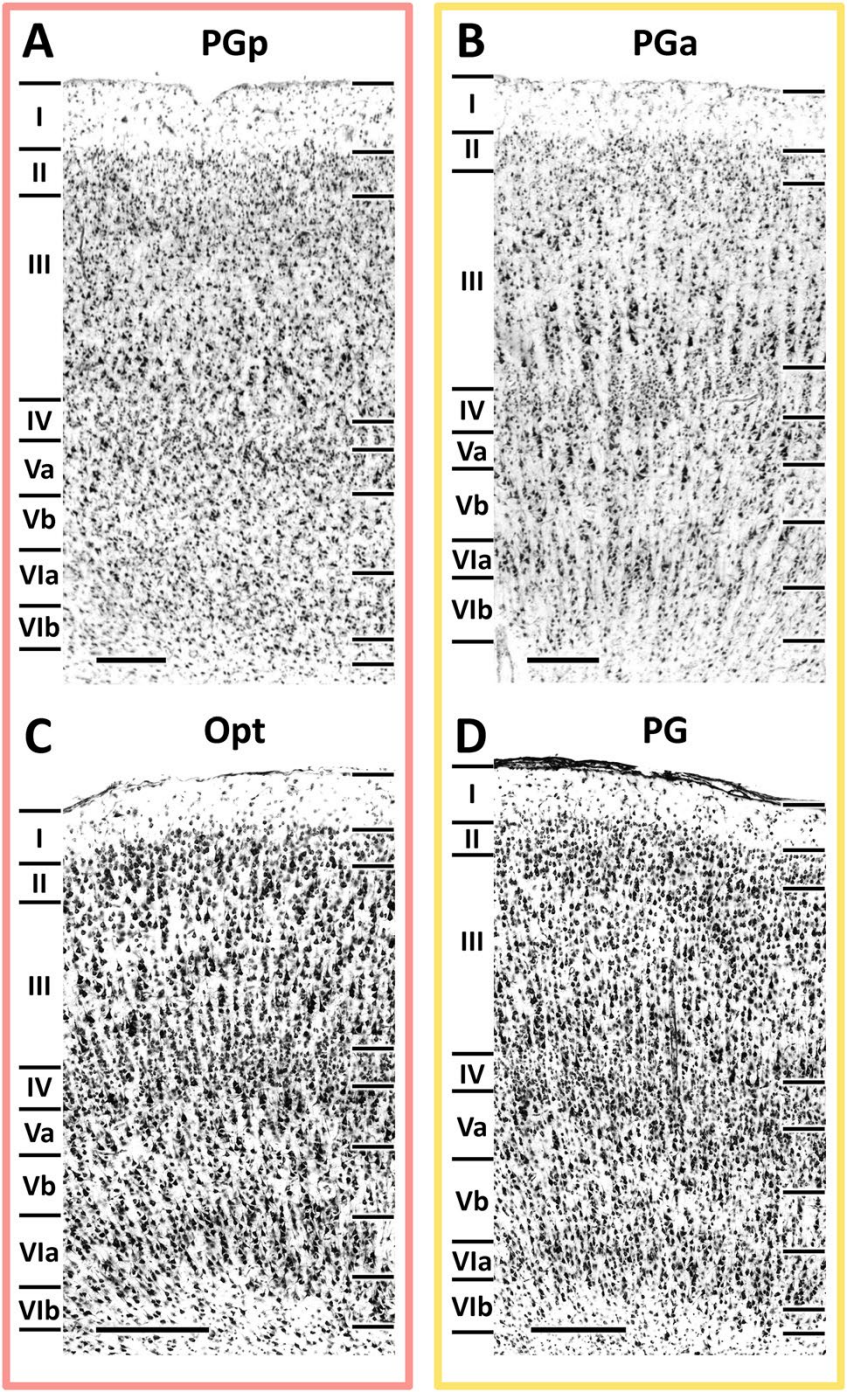
High-resolution photomicrographs of representative cytoarchitectonic fields through human areas PGp and PGa, and macaque areas Opt and PG. Scale bar, 300 μm. Roman numerals indicate cytoarchitectonic layers

As part of the isocortex, human areas PGp and PGa display the general cytoarchitecture of the six-layered cortex (Fig. 2A, B). Common cytoarchitectonic features of these two areas are the fact that i) layer II is difficult to separate from layer III since its granular cells intermingle with the small pyramids in upper layer III, and ii) layer IV is clearly detectable between layers III and V due to its homogeneous small-sized granule cells (Caspers et al. 2006). Layers V and VI can be subdivided into Va/b and VIa/b, respectively, in both the macaque (Niu et al. 2021) and the human brain. Although these sublayers had not been previously been defined in human areas PGp and PGa (Caspers et al. 2006), the comparative analysis in the present review led us to revise this lamination, since we found layers V and VI of human areas PGp and PGa to fulfill the criteria which resulted in their sublamination in areas Opt and PG, respectively, in the macaque brain.

Area PGp occupies the posterior portion of the human AG (Fig. 1). This area is characterized by an overall high packing density of relatively small-sized cells, and it has a relatively poorer lamination pattern than does PGa (Caspers et al. 2006). Layer III shows a minor superficial-to-deep increase in pyramidal cell size, with medium-sized pyramids spread in its lower one-third. It has a very narrow but clearly delineated layer IV. Layers V and VI contain comparably sized cells, but can be clearly separated by the lower cell density in Vb than in VIa. Although the Va/b and VIa/b borders are blurred, the border between layer VIb and the white matter is sharp (Fig. 2A, B).

Area PGa lies rostral to area PGp (Fig. 1) and is characterized by greater heterogeneities in both size and distribution of cell bodies (Caspers et al. 2006). It presents a clear lamination and a prominent columnar appearance, particularly in layers III, V and VI (Fig. 2B). PGa has a slightly thinner layer II and an overall lower cell-packing density in layer III than does area PGp. Furthermore, the size gradient found in layer III is more prominent in PGa than in PGp. Layer IV of PGa is somewhat broader than in PGp and shifts to a more superficial position in the cortex. Contrary to the homogeneity in PGp, the variation in cell size is quite evident in layers V and VI of PGa, with many relatively large-sized pyramids occupying their upper parts (i.e. Va and Via; Fig. 2A, B). Whereas the sublayer borders (i.e. the Va/b and VIa/b borders) are sharper in PGa than in PGp, the opposite holds true for the VIb/white matter border.

Areas PGp and PGa share common borders with the *ips* areas dorsally and the occipital lobe ventrally. Compared with *ips* areas, the cortex of AG areas becomes thicker and the cells of layer III decrease in size (Caspers et al. 2006). Ventrally, the cortex of the occipital lobe is relatively narrower and has larger pyramidal cells than those of PGp and PGa in lower layer III and layer V. The most important border in the IPL is the transition between its anterior and posterior part. Area PGa could be distinguished from anterior IPL by its more prominent layer IV, which becomes broader and shifts from the deepest third to the middle of the cortical width. Compared with anteriorly neighboring area PFm, there is a sudden increase in cell size in layer III of area PGa (Caspers et al. 2006).

In the macaque brain, areas Opt and PG are both characterized by a prominent layer IV, an overall clear lamination pattern, a columnar organization and a sharp layer VIb/white matter boundary (Niu et al. 2021; Fig. 2C, D). Caudal area Opt is further characterized by the presence of relatively large-sized neurons throughout all layers, as well as densely packed layers II and IV (Fig. 2C). Rostral area PG has considerably smaller, but more densely packed cells than Opt, and this is particularly true for layers III and VI (Fig. 2D). Layer III of PG presents a slight gradient in cell size, whereby the largest pyramids are located at the interface with layer IV. The border between layers V and VI is more prominent in PG than Opt due to the lower packing density in layer Vb of the former than the latter area.

As described for human areas PGp and PGa, macaque areas Opt and PG share common borders with the *ips* areas dorsally. They can be separated from *ips* areas by their more prominent layer IV, as well as by the larger cells in layer III. Ventrally, Opt and PG border with areas which encroach into the *sts* and lateral fissure, respectively. Caudal area Opt can be distinguished from posteriorly located parietal area PPt due to its broader cortical thickness and the palely stained layer III. Rostral area PG can be clearly delineated from parietal opercular area PGop due to the considerably poorer lamination in the infragranular layers of the latter area. Anteriorly, area PG can be clearly delineated from PFG because of its clearer lamination and less prominent columnar organization. Additionally, layer III of PG is thicker than that of PFG, and contains larger pyramids, particularly at the border with layer IV.

Summarizing, all four areas share the presence of i) a prominent layer IV, which is the main cytoarchitectonic feature enabling segregation of posterior from anterior areas in the IPL, and ii) a sublamination in layers V and VI. In both species, the cortex of the most posterior IPL area (i.e., human PGp and macaque Opt) is considerably thicker than that of the ventrally adjacent occipital cortex. Furthermore, Layer III in areas PGp and Opt is paler than that of layer III in the corresponding occipital area. Within the posterior IPL, layer Vb is more cell dense in the caudal areas of both species than in their rostral counterparts. However, despite these common features shared by both species, macaque areas Opt and PG seem to share more cytoarchitectonic similarities than do human areas PGp and PGa, since both macaque areas have a distinct lamination and columnar organization, but these characteristics are only present in human area PGa (Fig. 2).

### Myeloarchitecture

**Fig. 3:**
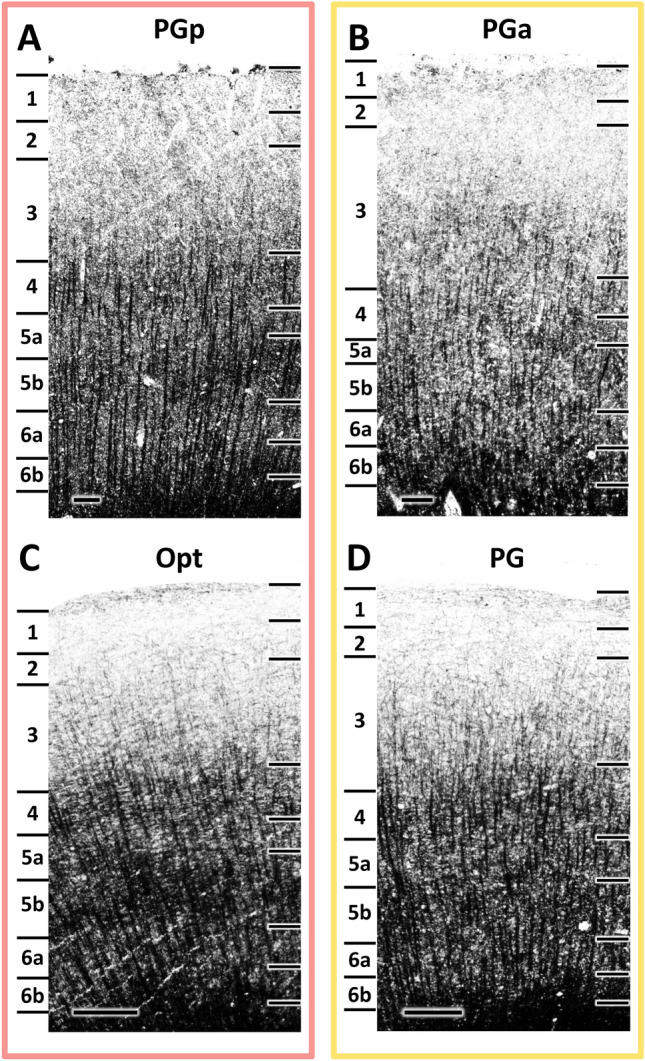
High-resolution photomicrographs of representative myeloarchitectonic fields through human areas PGp and PGa, and macaque areas Opt and PG. Scale bar, 300 μm. Arabic numerals indicate myeloarchitectonic layers

Similar to cytoarchitecture, human areas PGp and PGa as well as macaque areas Opt and PG share the basic myeloarchitectonic features of the isocortex, i.e., an euradiate structure. In both species, radial fiber bundles reach from the white matter up to the middle part of layer III. Furthermore, compared with sensory or modality-specific regions, all four areas are lightly myelinated, with only barely visible Baillarger stripes and thin radial fiber bundles (Fig. 3), and can thus be classified as being propeastriate (Vogt and Vogt 1919; Zilles et al. 2015).

Area PGp is characterized by a thin myeloarchitectonic layer (m-layer) 4 and a much broader and conspicuously pale m-layer 5 (Batsch 1956), which clearly separates the inner and outer bands of Baillarger from each other (Fig. 3A). Conversely, in PGa myeloarchitectonic layers are hardly delineable from each other (Batsch 1956; Fig. 3B).

In the macaque area, Opt, long, thick, vertically arranged bundles of fibers terminate within the deeper portion of m-layer 3 (Gregoriou et al. 2006; Lewis and Van Essen 2000). Both the inner and outer bands of Baillarger are prominent, mainly due to the pale staining in m-layer 5, since the horizontally and obliquely oriented nerve fibers are only barely visible (Fig. 3C).

Area PG presents an overall lower myelination than Opt, and both Baillarger bands are less prominent (Gregoriou et al. 2006). However, in PG radial fibers reach further m-layer 3 than they do in Opt. Vertical fiber bundles are visible; although they are thinner and less densely impregnated than those of Opt (Fig. 3D).

Summarizing, in both species the caudal areas (i.e., human PGp and macaque Opt) have a slightly higher degree of myelination than do the rostral areas (i.e., human PGa and macaque PG), particularly in the superficial layers. Furthermore, in both humans and macaques, the inner and outer Baillarger stripes are more prominent in the caudal than in the rostral areas. Thus, and in contrast to the situation described above for the cytoarchitecture, homolog areas between humans and macaques seem to share more common myeloarchitectonic features than do caudally versus rostrally located areas in each species.

### Receptor architecture

**Fig. 4:**
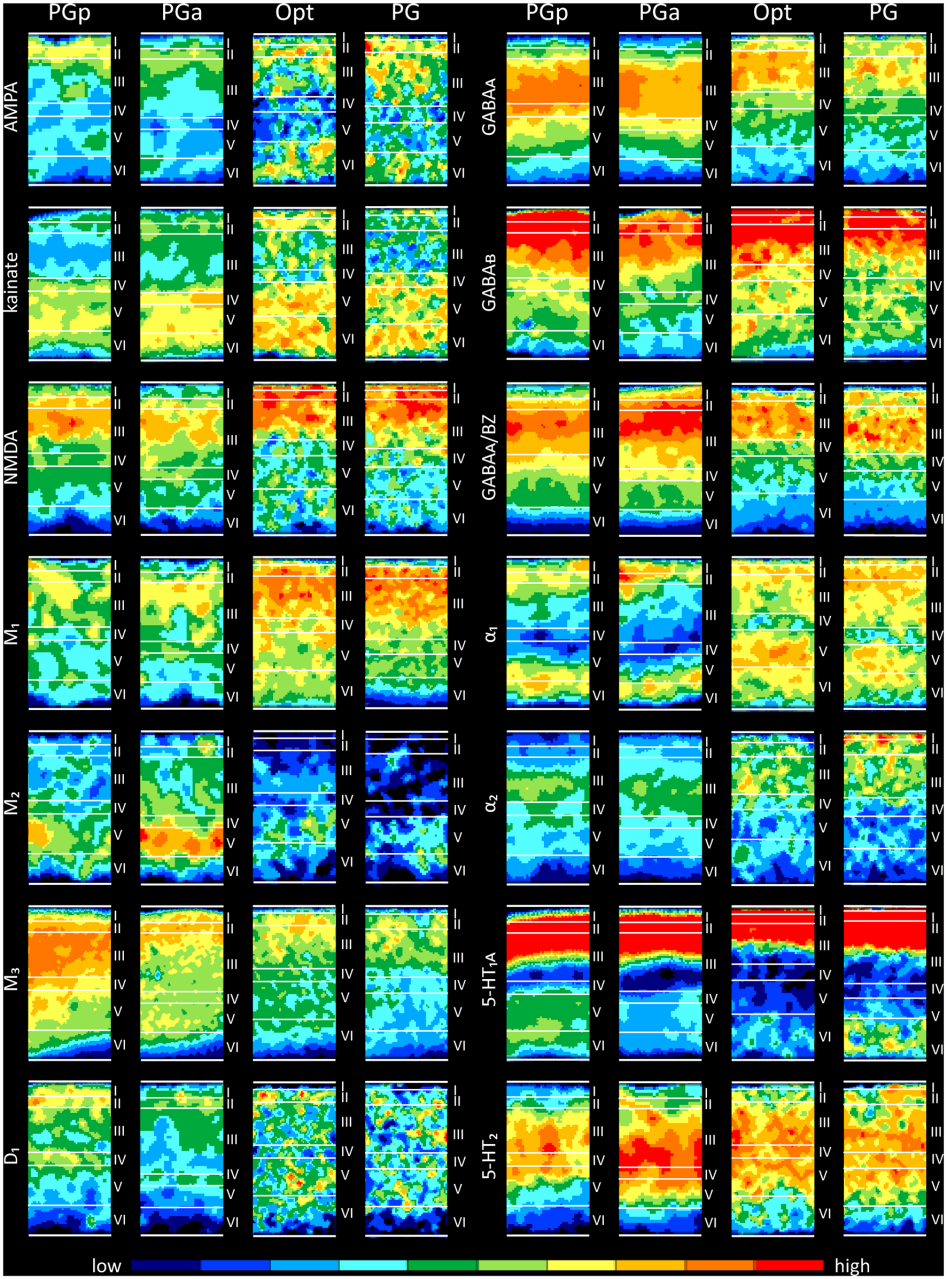
Laminar distribution of receptors for glutamate (AMPA, kainate, NMDA), GABA (GABA_A_, GABA_B_, GABA_A_/BZ), acetylcholine (M_1_, M_2_, M_3_), norepinephrine $(\alpha_{1} ,\,\alpha_{2} )$, serotonin (5-HT_1A_, 5-HT_2_), and dopamine (D_1_) in human areas PGp and PGa, and macaque areas Opt and PG. Color coding indicates receptor densities in fmol/mg protein. Blue tones indicate low densities and red tones high densities. For numeric information concerning receptor densities, see Table 2

Neurotransmitter receptors are heterogeneously distributed throughout the primate parietal lobe, and present distinct regional and laminar distribution patterns (Fig. 4) which enable the segregation of areas PGp and PGa in humans (Caspers et al. 2012; Zilles and Palomero-Gallagher 2001) and of areas Opt and PG in macaques (Geyer et al. 2005; Niu et al. 2021). In both species, absolute mean receptor concentrations vary considerably between the different receptor types in each area (Table 2). The highest absolute densities are found for the GABAergic receptors, followed by the glutamatergic, cholinergic, noradrenergic, serotonergic and dopaminergic receptors. Maximal values are reached by GABA_A_ associated benzodiazepine (GABA_A_/BZ) binding site in all areas, with the exception of macaque area PG, for which the highest densities were found for the GABA_B_ receptor. Lowest absolute densities are reached in all four areas by the D_1_ receptor (Table 2).

**Table 2:** Absolute receptor densities (mean ± sd in fmol/mg protein) of human areas PGp and PGa and their homologs in the macaque brain (Opt and PG, respectively)

	PGp	PGa	Opt	PG
AMPA	421 ± 176	377 ± 62	522 ± 108	571 ± 78
NMDA	1214 ± 306	1187 ± 395	1412 ± 187	1311 ± 278
Kainate	519 ± 397	553 ± 334	720 ± 96	674 ± 81
GABA_A_	1848 ± 482	1672 ± 524	1661 ± 226	1457 ± 282
GABA_B_	2303 ± 793	1889 ± 712	2049 ± 375	2067 ± 355
GABA_A_/BZ	3098 ± 1079	3349 ± 996	2152 ± 325	1942 ± 164
M_1_	621 ± 118	649 ± 95	995 ± 124	989 ± 111
M_2_	226 ± 59	227 ± 34	153 ± 44	132 ± 52
M_3_	1500 ± 121	1102 ± 321	831 ± 93	752 ± 104
α_1_	303 ± 149	299 ± 157	415 ± 39	434 ± 52
α_2_	159 ± 79	155 ± 51	289 ± 37	310 ± 44
5-HT_1A_	381 ± 185	369 ± 191	302 ± 59	388 ± 70
5-HT_2_	325 ± 144	382 ± 179	348 ± 87	342 ± 80
D_1_	84 ± 27	67 ± 31	87 ± 11	81 ± 9

AMPA receptors in human areas PGp and PGa present a unimodal distribution, with higher densities in the supragranular than in the infragranular layers. Area PGp reaches the highest density in layer II, whereas area PGa displays its maximal density at the border between layers II and III. The AMPA receptor distribution pattern in macaque areas Opt and PG is generically different from that of humans due to their bimodal laminar pattern. Area Opt reaches two maxima of comparable height, one in layers I–III, the second in layer VI, separated by low concentrations in layers IV–V. Area PG shows a superficial maximum in layer I–III and a second much lower one in layer VI. Kainate receptors in both human and macaque areas display a bimodal laminar distribution pattern. All areas present a maximum over layers IV–VI separated from the second maximum in layers I–II by conspicuously low densities in layer III. Interestingly, the two maxima reach comparable levels in macaque area Opt, whereas kainate receptor densities are considerably higher in the deep layers than in the superficial ones in macaque area PG and in human areas PGp and PGa. NMDA receptors show a unimodal distribution in all four areas, with the highest concentrations in the supragranular layers. NMDA receptor densities of the two human areas increase gradually through layers I–II, reach maximum values in upper layer III, and decrease again gradually through layers IV–VI. However, macaque areas Opt and PG present relatively narrow maxima, with the highest densities in layers I to II/III border, followed by much lower densities in the remaining layers.

GABAergic receptors present a unimodal laminar distribution pattern in areas PGp, PGa, Opt and PG, with higher densities in the supragranular than in the infragranular layers. However, the extent and localization of the absolute maxima differ between the different areas. Areas PGp and PGa display their highest GABA_A_ receptor densities in the lower part of layer III, whereas areas Opt and PG reach their maximal GABA_A_ concentrations in the upper part of layer III. No major differences were found in GABA_B_ receptor distribution patterns between areas PGp, PGa, Opt and PG, which present similar narrow maxima from layer I to the upper part of layer III. However, PGa could be distinguished from the other three areas by its significantly lower receptor densities, especially in the superficial layers. Regarding GABA_A_/BZ binding sites, areas PGp and PGa reach maximum densities in the upper part of layer III, followed by gradually declining concentrations from the lower part of layer III through to VIb. Conversely, macaque areas Opt and PG are characterized by high densities throughout layer III, and an abrupt decrease at the interface between layers III and IV followed by relatively low densities in layers V–VI.

The acetylcholine M_1_ and M_3_ receptors display a unimodal distribution of their laminar concentrations in all four areas, with the highest densities in the superficial half of the cortical ribbon. For human areas PGp and PGa, after the maximum in the supragranular layers, M_1_ receptors show a relatively sharp decline when moving towards the layer VI/white matter boundary. In contrast, macaque area Opt and PG display a shallower decrease starting from the lower part of layer III. The opposite holds true for M_3_ receptors. I.e., the decrease in M_3_ receptor densities when moving from the superficial layers to the layer VI/white matter boundary follows a steeper course in macaque areas Opt and PG than in human areas PGp and PGa. A notable laminar distribution pattern is shown by the M_2_ receptor, which displays a very distinct and restricted distribution of relatively high concentrations in layer V. This local maximum is more pronounced in areas of the human brain than in macaque areas Opt or PG.

Adrenergic α_1_ receptors present a bimodal distribution pattern in all four areas, though they differ in the width and location of the maxima. Human areas PGp and PGa present two relatively narrow maxima of comparable height, and separated by a broad band of low concentrations encompassing layers III-Va. Macaque areas Opt and PG also present two maxima of comparable height, though they are relatively broad and separated by a very narrow minimum restricted to layer IV. The laminar distribution pattern of α_2_ receptors also differs considerably between human and macaque areas. Human areas PGp and PGa both present a relatively restricted maximum in the lower part of layer III. Macaque areas Opt and PG show a superficial maximum in layer III and I/II, respectively, followed by abruptly lower densities as from layer IV.

Human and macaque areas present a similar bimodal 5-HT_1A_ receptor distribution pattern, with a prominent superficial maximum in layers I to upper III, a single sharp minimum around layer IV, and a second, shallower maximum in the deep layers. Human areas could be distinguished from their homologs in the macaque brain mainly by the location and extent of the second maximum, which is located over layers V–VI of PGp and PGa, but restricted to layer VI in Opt and PG. Interestingly, whereas in humans the deeper maximum is more pronounced in caudal area PGp than in rostral area PGa, in macaques the opposite holds true, and layer VI of PG presents higher 5-HT_1A_ receptor densities than that of Opt. Serotonergic 5-HT_2_ receptors present a wide maximum in areas PGp and PGa, with high densities through layers III–Va, which is flanked by relatively low densities in the remaining layers. Macaque area Opt presents a comparable laminar distribution pattern, but in PG low 5-HT_2_ receptor densities are restricted to layer VI.

The dopaminergic D_1_ receptors are bimodally distributed in human areas PGp and PGa, with a first maximum in I/II border and a second, much lower one in layer IV. The macaque posterior IPL presents a considerably lower D_1_ receptor density then human one. Furthermore, D_1_ receptors are homogeneously distributed throughout all layers of areas Opt and PG.

From an overall perspective, the inter-species variations are more evident than the inter-areal differences within each species, mainly due to differences in receptor densities averaged over all layers, whereby macaque areas showed generally higher concentrations of more examined receptors than human areas. Despite these differences, there are important similarities between homolog areas of the human and macaque posterior IPL. For example, both species mainly present comparable laminar distribution patterns, with higher densities for most receptor types in the supragranular than the infragranular layers. Importantly, both species share in common the fact that the absolute densities of most receptor types decreased when moving from the caudal to rostral within the IPL.

## Structural and functional connectivity patterns

### Histological tract tracing

This section will start with a description of macaque connectivity, since tracer studies in human brains have only been rarely performed (Lim et al. 1997), and to our knowledge none exist for the AG. Using invasive tracer techniques, the connectivity patterns of the posterior IPL areas in the monkey have been studied extensively (Andersen et al. 1990; Cavada and Goldman-Rakic 1989a, b; Felleman and Van Essen 1991; Mesulam et al. 1977; Neal et al. 1990; Petrides and Pandya 1984; Rozzi et al. 2006; Schmahmann and Pandya 2006). This section provides an overview of the major results to allow for comparisons with the indirect measure of structural connectivity obtained in humans via diffusion imaging.

In the monkey brain, both Opt and PG show strong connections to their surrounding areas, which are located in the IPL, *ips,* parieto-occipital junction and posterior *sts* (Andersen et al. 1990; Caspers et al. 2011; Cavada and Goldman-Rakic 1989a, b; Katsuyama et al. 2010; Mesulam et al. 1977; Petrides and Pandya 1984; Rozzi et al. 2006). Specifically, Opt shows very strong and consistent connections via U-fibers with areas PG and PGm, areas in the posterior part of *ips*, as well as areas within the upper bank of posterior *sts*. Additionally, Opt shows a relatively weak connection with the parietal operculum (area PGop), *ips* areas MIP and VIP. The most significant connections of area PG were observed with IPL areas PFG, PGop, and the rostral part of area Opt. Moderate connections can be also found between PG with *ips* areas MIP, AIP, and cingulate area 23, PEci.

Furthermore, long association connections of the caudal IPL are directed to the frontal lobe, the cingulate gyrus, the multimodal areas of the temporal lobe, and the parahippocampal gyrus (Andersen et al. 1990; Caspers et al. 2011; Cavada and Goldman-Rakic 1989a, b; Luppino et al. 2001; Mesulam et al. 1977; Petrides and Pandya 1984; Rozzi et al. 2006; Schmahmann and Pandya 2006; Schmahmann et al. 2007). Caudal IPL is anatomically connected mainly via five association fibers to other cortical areas: the second branch of the Superior Longitudinal Fasciculus (SLF II), the Middle Longitudinal Fasciculus (MdLF), the Inferior Longitudinal Fasciculus (ILF), the Fronto-Occipital Fasciculus (FOF) and the Cingulum Bundle (CB) (Schmahmann and Pandya 2006; Schmahmann et al. 2007). SLF II interlinks caudal IPL with caudal prefrontal areas 46, 9/46, 8Ad and 6D (Petrides and Pandya 1984, 2006). MdLF links caudal IPL with the cingulate, prefrontal, superior temporal regions and the parahippocampal gyrus (Petrides and Pandya 1984, 2006). ILF connects the most caudal part of IPL (e.g. Opt) with the occipital, multiple inferior temporal regions and extends to the temporal pole (Tusa and Ungerleider 1985). Similar to SLF II, the FOF also links the caudal IPL with frontal lobe areas (Schmahmann and Pandya 2007). The CB fibers extend rostrally into the lateral and medial frontal lobes, and caudally into the parietal lobe. CB fibers leading to the parietal lobe arise from, or terminate in, areas Opt/PG and medially in retrosplenial areas 29 and 30 (Schmahmann and Pandya 2006; Schmahmann et al. 2007). Due to the existence of these long association fibers, Opt shows strong connections with the parahippocampal gyrus, particularly areas TF and TH. Moderate connections were observed between Opt and the higher visual areas, as well as with the upper (area STP) and ventral (area TE) banks of the *sts*. In the frontal lobe, Opt connected with premotor areas F5, F7, subdivisions of prefrontal area 46, and frontal eye-related areas (8A and 8B). PG has strong connections with the insular and retroinsular cortex, and also more rostrally and deeply, with area SII. Furthermore, area PG is connected with premotor areas F2, F5, and prefrontal area 46v.

### Non-invasive assessment of structural connectivity

More recently, the development of MRI diffusion tractography has allowed for the visualization of white matter tracts that link macaque caudal IPL to other brain regions. Most of the findings from the tract-tracing studies have been further clarified by diffusion tensor imaging (DTI) and diffusion spectrum imaging (DSI) studies (Barrett et al. 2020; Calabrese et al. 2015; Feng et al. 2017; Hofer and Frahm 2008; Makris et al. 2005; Schmahmann et al. 2007; Thiebaut de Schotten et al. 2012; Zakszewski et al. 2014).

Furthermore, by using these techniques, several local U-shaped connections were identified in macaque brains (Catani et al. 2017): Parietal Inferior-to-Superior Tract (PIST), Parietal angular-to-supramarginal (PAS), Parietal Intra-Gyral tract of the supramarginal gyrus (PIG-SMG). PIST is a vertical pathway between the superior and inferior parietal lobules. It can be divided into a posterior (PIST-AG) and an anterior (PIST-SMG) parts. The PIST-AG connects Opt to PEc in the SPL, whereas the PIST-SMG connects PG to areas PE and PEc. PAS is a small U-shaped tract between areas DP/Opt and PG, PIG-SMG allows communication between PG to rostral IPL areas PFG and PF (Catani et al. 2017).

Simultaneously, white matter connections in humans have also been demonstrated through DTI/DSI studies (Barrett et al. 2020; Caspers et al. 2011; Catani et al. 2005; Frey et al. 2008; Makris et al. 2005; Menjot de Champfleur et al. 2013; Rilling et al. 2008; Ruschel et al. 2014; Thiebaut de Schotten et al. 2012; Uddin et al. 2010). As in the macaque brain, the SLF II connects the AG to the caudo-lateral prefrontal regions (Makris et al. 2005). Moreover, the third branch of the SLF (SLF III) links the AG directly to the inferior frontal gyrus at the level of areas BA 44 (Frey et al. 2008) and BA 45 in the human brain. Human AG is also connected to the caudal posterior temporal regions via the MdLF (Frey et al. 2008; Menjot de Champfleur et al. 2013) and to both the parahippocampal gyrus (Rushworth et al. 2006) and hippocampus (Uddin et al. 2010) via the ILF. The FOF links the AG with the precuneus (BA 7) and the superior frontal gyrus (BA 8). Furthermore, human AG is additionally connected to other cortical areas via the Arcuate Fasciculus (AF) and the Inferior Fronto-Occipital Fasciculus (IFOF), which seem to be unique to humans. Both PGp and PGa are connected to the caudate via the IFOF (Uddin et al. 2010). The AF runs from the inferior frontal gyrus via the AG (i.e., the posterior IPL) to the superior temporal cortex. Although the long segment of AF avoids the AG, one of the short segments enable connections between the AG and the superior temporal cortex (Catani and De Schotten 2008; Catani et al. 2005; Rilling et al. 2008). Although the AF has also been described in the non-human primate brain, in macaques it is proportionally smaller and less well developed than in humans (Eichert et al. 2019; Rilling et al. 2008). Furthermore, in macaques the AF connects ventrolateral prefrontal areas 44 and 45 with the anterior IPL (area 7b, which encompasses areas PF and PFG of Niu et al. (2021)) and area Tpt in the posterior *sts* (Rilling et al. 2008; Yeterian et al. 2012).

Although the short intralobar tracts are mostly conserved between humans and monkeys, interspecies differences are evident for some tracts (Catani et al. 2017). In humans, PIST-SMG and PIG-SMG do not pass through AG, and only PIST-AG links both PGp and PGa with the superior parietal lobule. There are also differences in the PAS since in humans it passes under the Jensen sulcus and connects PGa with the posterior supramarginal gyrus, but in macaque this tract runs between Opt and PG. Furthermore, PIP-AG, a human-specific tract, is one of the branches of the Parietal inferior-to-postcentral (PIP) tract (Catani et al. 2017). The PIP-AG projects mainly to the handknob region of the postcentral gyrus and the dorsal region of the angular gyrus after passing beneath the postcentral and intraparietal sulci (Catani et al. 2017).

For the human brain, differential fiber densities when moving from caudal to rostral areas of the AG have also been identified: caudal area PGp shows more connections to posterior parts of the superior parietal, temporo-occipital, lateral occipital, auditory as well as inferior frontal cortices. Moreover, PGp shows a greater density of fibers connecting to the hippocampus and parahippocampal region than does PGa. Whereas PGa shows stronger connections to somatosensory and superior parietal cortices, and additionally features consistent fiber tracts to premotor and ventral prefrontal cortices (Uddin et al. 2010; Wang et al. 2016).

### Functional connectivity

From the functional connectivity perspective, macaque caudal IPL areas show comparatively strong connectivity with the dorsal prefrontal cortex and areas around the arcuate sulcus (Mars et al. 2011, 2013; Vijayakumar et al. 2019). Specifically, Opt is most strongly connected to the parahippocampal cortex and area 9. PG can be separated from Opt due to its stronger connectivity with the peri-arcuate areas (area 8, 44, 45). Moreover, PG shows extra connectivity with F7, F2 (Mars et al. 2011, 2013; Vijayakumar et al. 2019).

In the human brain, AG areas show a consistent functional connectivity with the corresponding areas in the contralateral hemisphere, and with the posterior cingulate, precuneus, inferior temporal gyrus, dorsolateral, ventral, medial prefrontal cortices and the frontal pole (Kelly et al. 2010; Mars et al. 2011; Uddin et al. 2010; Wang et al. 2017, 2016). Direct comparisons of the PGp and PGa revealed greater connectivity of PGp with bilateral parahippocampal and hippocampal gyri, and with the adjacent lateral occipital cortex (Uddin et al. 2010). PGa shows greater connectivity than PGp with the caudate nucleus, the frontal pole, and the cingulate gyrus (Kelly et al. 2010; Mars et al. 2011; Uddin et al. 2010; Wang et al. 2017, 2016).

Summarizing, although the human and macaque posterior IPL areas share numerous similarities in their connectivity patterns, there are also some differences between these two species. Similarities include the connections of posterior IPL areas with other brain regions via the SLF II, MdLF, ILF and FOF. A notable difference is the AF, which is greatly expanded in the human brain in comparison with the macaque brain (Rilling et al. 2008). Furthermore, the connectivity pattern in human AG areas shows prominent lateralization patterns, which has not been observed in the connectivity of macaque posterior IPL areas (Caspers et al. 2011; Wang et al. 2016, 2017).

## Functional considerations

Functionally, the caudal IPL is commonly considered as part of the heteromodal parietal association cortex. In both humans and macaque monkeys, the posterior IPL is mainly involved in multisensory integration and transformation of sensory information into the guidance of motor behavior (Yokoyama et al. 2021). In addition, this region also subserves spatial functions, including general spatial perception, estimation of directions, processing of extrapersonal and peripersonal space, as well as the localization of objects (Binkofski et al. 2016; Buckner et al. 2008; Rozzi et al. 2008; Seghier 2013).

Specifically, human area PGp and macaque area Opt have been associated with tasks related to visual perception, attentional shifts, grasping, effecting strategy and memory retrieval (Mesulam 1999; Nelson et al. 2010; Rozzi et al. 2008; Sharp et al. 2010; Simon et al. 2002). Interestingly, in both humans and macaque monkeys, these different functions are processed following a gradient along the IPL caudo-rostral axis (Caspers et al. 2011; Rozzi et al. 2008; Uddin et al. 2010). These two caudal areas are largely connected to the extrastriate visual cortex and temporal visual areas (Caspers et al. 2011; Mars et al. 2011; Rozzi et al. 2006), so they are generally considered to be visually responsive areas, and they seem to be a component of the dorsal visual stream for the transformation of visual input to visual associations (Caspers et al. 2012; Niu et al. 2021). Additionally, caudal areas are involved in space perception and the guidance of motor behavior, particularly in the control of saccadic (Barash et al. 1991) and oculomotor (Bremmer et al. 1997; Bruce et al. 1985) movements. This fact is also supported by studies showing that human area PGp and macaque area Opt are connected with the functionally defined frontal eye-related areas (Caspers et al. 2011; Rozzi et al. 2006).

In contrast, human PGa and macaque PG seem to be mostly involved in the integration of multisensory information, rather than in visual input transformation. In both species, these two areas are extensively connected with visual areas of both the dorsal and the ventral visual streams (Caspers et al. 2011; Ilg and Schumann 2007; Luppino et al. 2001; Rozzi et al. 2006; Uddin et al. 2010). Moreover, areas PGa and PG receive somatosensory information from SII and retroinsular cortex (Caspers et al. 2011; Murray and Coulter 1981; Robinson and Burton 1980), as well as auditory information from auditory areas in the temporal lobe (Mars et al. 2011; Morel et al. 1993). Furthermore, the rostral areas are more strongly involved in the control of arm movements than in eye movements (Andersen et al. 1990; Hyvärinen 1981), which is consistent with connectional studies (Caspers et al. 2011; Pyke et al. 2015; Rozzi et al. 2006) showing that rostral areas are connected with the functionally defined arm-related areas (Ilg and Schumann 2007; Luppino et al. 2001).

In addition to multisensory integration and spatial functions, human AG areas are also involved in higher-order cognitive processes such as decoding the meaning of personal morally relevant interactions, being particularly concerned with egocentric and allocentric perspective taking (Buckner et al. 2008; Raine and Yang 2006; Spreng et al. 2009), as well as language (Dronkers et al. 2004; Humphreys et al. 2021), speech (Hartwigsen et al. 2015; Obleser and Kotz 2010), reading, verbal working memory (Hutchinson et al. 2009; Van Dam et al. 2015), and number processing (Göbel et al. 2001; Pyke et al. 2015). Indeed, semantic processing, i.e., retrieval of word meanings is the most consistent function associated with that activations of the human AG (Binder et al. 2009; also see articles by Desai et al., Kuhnke et al., Graves et al., Sokolowski et al., Pinheiro-Chagas and Parvizi in the present Special Issue). In the human brain, these processes are thought to rely on the activation of brain areas occupying the highest hierarchical positions in multimodal networks, as are PGp and PGa (Binder and Fernandino 2015; Margulies et al. 2016; Xu et al. 2020). It is debated whether the hierarchical position of macaque posterior IPL areas is as high as that of human AG areas (Binder and Fernandino 2015; Xu et al. 2020).

Taken together, the functional evidence concerning the role of posterior IPL areas in multisensory integration and in the modulation of visuo-motor behavior further supports the homology between human areas PGp and PGa with monkey areas Opt and PG, respectively. However, further research is necessary to fully understand the involvement of macaque posterior IPL areas in higher cognitive functions.

## Summary

Macaque monkey models are necessary to gain a comprehensive understanding of the relationship between the brain’s structural and functional segregation, as well as to accurately simulate the pathogenic, histological, biochemical, or clinical features of neuropsychiatric and neurological diseases. This review highlights how the contrast between the simplicity of macaque gross anatomy and the particularly high complexity and variability of sulcal patterns in the human posterior IPL has hindered the identification of homolog areas in this brain region. Further, it provides a comprehensive overview of the cyto-, myelo- and receptor architectonic features as well as connectivity patterns of human areas PGp and PGa and macaque areas Opt and PG with a focus on assessing the (dis)similarities which provide insights into establishing homologies between human areas and their macaque counterparts. We found striking comparability of microarchitectonic and connectivity features between human and macaque areas in the posterior IPL. Although species differences were found for receptor densities averaged over all cortical layers, comparable trends in the rostro-caudal gradations in receptor densities and similarities in laminar distribution patterns support the definition of macaque areas Opt and PG as homologs of human areas PGp and PGa, respectively.

## Data Availability

The authors confirm that the data supporting the findings of this study are available within the article.
